# Fosfomycin for Injection (ZTI-01) Versus Piperacillin-tazobactam for the Treatment of Complicated Urinary Tract Infection Including Acute Pyelonephritis: ZEUS, A Phase 2/3 Randomized Trial

**DOI:** 10.1093/cid/ciz181

**Published:** 2019-03-06

**Authors:** Keith S Kaye, Louis B Rice, Aaron L Dane, Viktor Stus, Olexiy Sagan, Elena Fedosiuk, Anita F Das, David Skarinsky, Paul B Eckburg, Evelyn J Ellis-Grosse

**Affiliations:** 1 Division of Infectious Diseases, University of Michigan Medical School, Ann Arbor; 2 Department of Medicine, Warren Alpert Medical School of Brown University and Rhode Island Hospital and The Miriam Hospital, Providence; 3 DaneStat Consulting, Alderly Edge, United Kingdom; 4 Municipal Institution Dnipropetrovsk Medical Academy of Ministry of Health of Ukraine, Dnipro; 5 Municipal Institution Zaporizhzhia Regional Clinical Hospital of Zaporizhzhia, Regional Council Department of Urology, State Institution Zaporizhzhia Medical Academy of Postgraduate Education under the Ministry of Health of Ukraine; 6 Brest Regional Hospital, Belarus; 7 Das Statistical Consulting, Guerneville; 8 Zavante Therapeutics, Inc., San Diego, California

**Keywords:** ZTI-01, fosfomycin, complicated urinary tract infection, acute pyelonephritis

## Abstract

**Background:**

ZTI-01 (fosfomycin for injection) is an epoxide antibiotic with a differentiated mechanism of action (MOA) inhibiting an early step in bacterial cell wall synthesis. ZTI-01 has broad in vitro spectrum of activity, including multidrug-resistant Gram-negative pathogens, and is being developed for treatment of complicated urinary tract infection (cUTI) and acute pyelonephritis (AP) in the United States.

**Methods:**

Hospitalized adults with suspected or microbiologically confirmed cUTI/AP were randomized 1:1 to 6 g ZTI-01 q8h or 4.5 g intravenous (IV) piperacillin-tazobactam (PIP-TAZ) q8h for a fixed 7-day course (no oral switch); patients with concomitant bacteremia could receive up to 14 days.

**Results:**

Of 465 randomized patients, 233 and 231 were treated with ZTI-01 and PIP-TAZ, respectively. In the microbiologic modified intent-to-treat (m-MITT) population, ZTI-01 met the primary objective of noninferiority compared with PIP-TAZ with overall success rates of 64.7% (119/184 patients) vs 54.5% (97/178 patients), respectively; treatment difference was 10.2% (95% confidence interval [CI]: −0.4, 20.8). Clinical cure rates at test of cure (TOC, day 19–21) were high and similar between treatments (90.8% [167/184] vs 91.6% [163/178], respectively). In post hoc analysis using unique pathogens typed by pulsed-field gel electrophoresis, overall success rates at TOC in m-MITT were 69.0% (127/184) for ZTI-01 versus 57.3% (102/178) for PIP-TAZ (difference 11.7% 95% CI: 1.3, 22.1). ZTI-01 was well tolerated. Most treatment-emergent adverse events, including hypokalemia and elevated serum aminotransferases, were mild and transient.

**Conclusions:**

ZTI-01 was effective for treatment of cUTI including AP and offers a new IV therapeutic option with a differentiated MOA for patients with serious Gram-negative infections.

**Clinical Trial Registration:**

NCT02753946

## Teaser

ZEUS, a Phase 2/3 trial, studied ZTI-01 (fosfomycin for injection) in the treatment of hospitalized adults with complicated urinary tract infection and acute pyelonephritis versus piperacillin-tazobactam. ZTI-01 was non-inferior to piperacillin-tazobactam and was well tolerated.

**(See the Editorial Commentary by Harris on pages 2057–8.)**

Prevalence of drug resistance has increased steadily resulting in the need for safe and effective antibiotic treatment options, particularly for multidrug-resistant (MDR) Gram-negative bacteria [1]. As rates of serious infections intensify, clinicians are forced to use agents of last resort or agents associated with toxicity (eg, polymyxins, aminoglycosides). Although some new β-lactam agents with activity against MDR pathogens have recently received Food and Drug Administration (FDA) approval, there is a need for additional options with differentiated mechanisms of action (MOAs) that are safe and effective. Alternatives to β-lactams are needed due to class-associated safety concerns (eg, hypersensitivity) and/or rapidly emerging resistance [2].

ZTI-01 (fosfomycin for injection) is an injectable epoxide and sole antibiotic class member. The differentiated MOA inhibits an early step in peptidoglycan biosynthesis by covalently binding to MurA [3]. In in vitro studies, ZTI-01 has demonstrated a broad spectrum of activity against a variety of clinically important MDR Gram-negative pathogens (including extended-spectrum beta-lactamase [ESBL]-producing Enterobacteriaceae and carbapenem-resistant Enterobacteriaceae [CRE]) and Gram-positive pathogens (including methicillin-resistant *Staphylococcus aureus* and vancomycin-resistant enterococci) [4].

In the United States, a tromethamine salt of fosfomycin is available, administered as a single 3-gram oral sachet indicated for treatment of uncomplicated urinary tract infection (UTI, ie, cystitis) [5]. For more serious infections, oral administration provides inadequate concentrations due to its limited bioavailability (37%) and dose-limiting gastrointestinal tolerability [5].

Outside the United States, intravenous (IV) fosfomycin, as evidenced in >40 years of use in more than 60 clinical studies, has provided a safe and effective treatment option for patients with a variety of infections, often severe (ie, complicated UTI [cUTI], bacteremia, acute osteomyelitis, nosocomial pneumonia, surgical site infections, bone and joint infections, endocarditis, complicated skin infections, and bacterial meningitis) [3, 6]. Fosfomycin retains high in vitro activity with low and stable resistance despite longevity of use.

ZTI-01 is being developed for treatment of cUTI/acute pyelonephritis (AP) in the United States. This report presents the efficacy and safety results from the ZEUS trial, a multicenter, randomized, double-blind, phase 2/3 trial designed to evaluate safety and efficacy of ZTI-01 in treating hospitalized adults with cUTI/AP versus piperacillin-tazobactam (PIP-TAZ).

## METHODS

### Objectives

The primary objective was to demonstrate noninferiority (NI) of ZTI-01 to PIP-TAZ in overall success (clinical cure and microbiologic eradication) in microbiologic modified intent-to-treat (m-MITT) population at test of cure (TOC, day 19–21). Secondary objectives were to compare: (1) clinical cure rates in the 2 treatment groups in MITT, m-MITT, clinical evaluable (CE), and microbiologic evaluable (ME) populations at TOC, and (2) microbiological eradication rates in m-MITT and ME populations at TOC.

### Study Design and Participants

**Figure 1.**
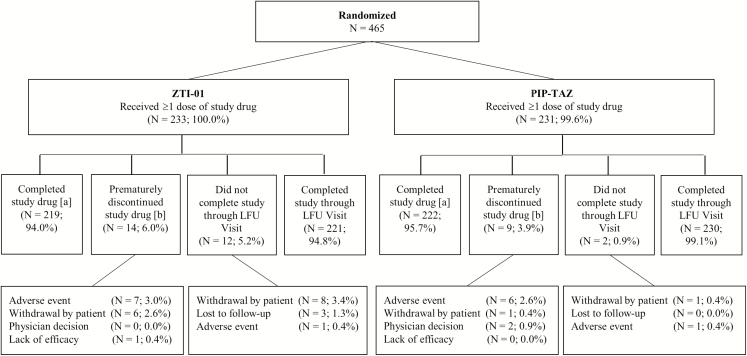
Study design. N: Number of patients in the ITT population. Percentages were calculated using the number of patients in the ITT population as the denominator. *A*, Completed study drug was defined as completing 7 to 14 days of treatment. *B*, Only included patients who received study drug. Patient 1401–16 had an adverse event and did not receive study drug. Abbreviations: ITT, intent-to-treat; LFU, late follow-up; PIP-TAZ, piperacillin-tazobactam; TOC, test of cure; ZTI-01, fosfomycin for injection.

ZEUS was a multicenter, randomized, parallel-group, double-blind phase 2/3 trial designed to evaluate safety, tolerability, efficacy, and pharmacokinetics of ZTI-01 in treating hospitalized adults with cUTI/AP versus PIP-TAZ at 92 global sites in 16 countries (Figure 1).

Patients provided written informed consent. The study was conducted according to the International Conference on Harmonisation Good Clinical Practice guidelines, Directive 2001/20/EC, applicable regulatory requirements, and the Declaration of Helsinki [7]. Protocol was approved by local health agencies, study sites’ independent ethics committees, and/or institutional review boards (clinicaltrials.gov NCT02753946). An independent, blinded, data monitoring committee assessed ZTI-01 safety and baseline evaluability for sample size adjustment (m-MITT).

The Supplementary Appendix lists full inclusion/exclusion criteria.

### Randomization and Masking

Eligible patients were randomized 1:1 to receive either 6 g ZTI-01 infused over 60 minutes every 8 hours [q8h] or 4.5 g PIP-TAZ (4 g PIP/0.5 g TAZ) infused over 60 minutes q8h. The PIP-TAZ dosage was based on labeled guidelines for cUTI/AP in the current Summary of Medicinal Product Characteristics [8], with no dosage adjustment necessary. ZTI-01 doses were adjusted for patients with CrCl <50 and ≥20 mL/min (Supplementary Appendix). Subjects with baseline CrCl <20 mL/min were excluded from the study. Randomization was stratified by region (US vs Rest of World), baseline diagnosis (cUTI vs AP), and prior antibiotic therapy (single dose of short-acting antibiotic vs no prior antibiotic therapy). At least 30% of patients were intended to have a diagnosis of AP at study entry.

Patients received ZTI-01 and PIP-TAZ as 1-hour infusions 3 times daily for a fixed 7 days, except in patients with concurrent bacteremia who could receive up to 14 days at the investigator’s discretion. Oral step-down therapy was prohibited.

### Study Procedures

Procedures included baseline urine collection for quantitative culture and blood cultures. Routine monitoring occurred for signs and symptoms of cUTI/AP and adverse events (AEs), and prespecified collection of laboratory data including chemistry panels, complete blood counts, and urine/blood cultures (Supplementary Appendix).

Baseline urine cultures growing ≥10^5^ colony-forming units (CFU)/mL and any positive blood cultures were sent to the central laboratory (JMI Laboratories, North Liberty, Iowa) for identification, quantification, susceptibility testing, and further characterization of the organism(s). Fosfomycin minimum inhibitory concentrations (MICs) were determined at a central lab using the agar dilution reference method.

### Efficacy Endpoints

Primary endpoint of overall success was a composite of investigators’ determination of clinical cure (complete resolution or significant improvement of signs and symptoms such that no further antimicrobial therapy is warranted) plus microbiologic eradication (baseline pathogen was reduced to <10^4^ CFU/mL on urine culture and if applicable, negative on repeat blood culture) in the m-MITT at TOC. Subjects with any missing postbaseline urine sample were classified as indeterminates, and conservatively deemed as failures in overall success analysis. Secondary efficacy endpoints included proportion of patients with clinical cure in MITT, m-MITT, and CE, and ME groups at TOC, and proportion of patients with microbiologic eradication in m-MITT and ME groups at TOC.

Pathogens isolated from patients who had a baseline and TOC pathogen underwent blinded, post hoc, pulsed-field gel electrophoresis (PFGE) typing. Microbiologic outcome was defined utilizing PFGE results whereby microbiologic persistence required the same genus and species of baseline and postbaseline pathogens as well as PFGE-confirmed genetic identity.

### Phenotypic Resistance

Using MICs, blood or urine pathogens resistant to other antibiotic classes were categorized as: ESBL production (aztreonam, ceftazidime, or ceftriaxone MIC ≥2 µg/mL), CRE (imipenem or meropenem MIC ≥4 µg/mL), aminoglycoside resistance (gentamicin ≥8 µg/mL or amikacin ≥32 µg/mL), or MDR (nonsusceptibility to ≥3 antibiotic classes). Patients with multiple organisms of the same phenotype were counted only once.

### Safety

Patients who received any amount of study drug were included in the safety population. Safety endpoints included assessment of treatment-emergent AEs (TEAEs) and evaluation of changes from baseline in laboratory test results, ECGs and vital signs. AEs were coded using version 19.0 of Medical Dictionary for Regulatory Activities (MedDRA). Hy’s law was defined as alanine aminotransferase (ALT) or aspartate aminotransferase (AST) >3 × upper limit of normal (ULN), alkaline phosphatase (ALP) ≤2 × ULN, and total bilirubin >2 × ULN.

### Statistical Analyses

Sample size of 230 patients per arm (N = 460) was based on FDA-agreed upon 15% NI margin, 70% predicted evaluability rate, 70% overall success rate in both treatment groups, 80% power, and 1-sided α = 0.025. Number and percentage of patients in each treatment group with an overall success, failure, and indeterminate response (ie, patients with missing data) were determined and response rate for the primary analysis was defined as the number of successes divided by the number of success, failure, and indeterminate. A 2-sided 95% confidence interval (CI) for the observed difference in overall success rate (ZTI-01 group minus PIP-TAZ group) was calculated using a continuity-corrected Z-statistic. Noninferiority of ZTI-01 to PIP-TAZ determined if the lower limit of the 95% CI for the difference in the m-MITT was >−15%. Analyses were performed using SAS version 9.1 or higher (SAS Institute, Cary, North Carolina).

## RESULTS

### Patients

**Figure 2.**
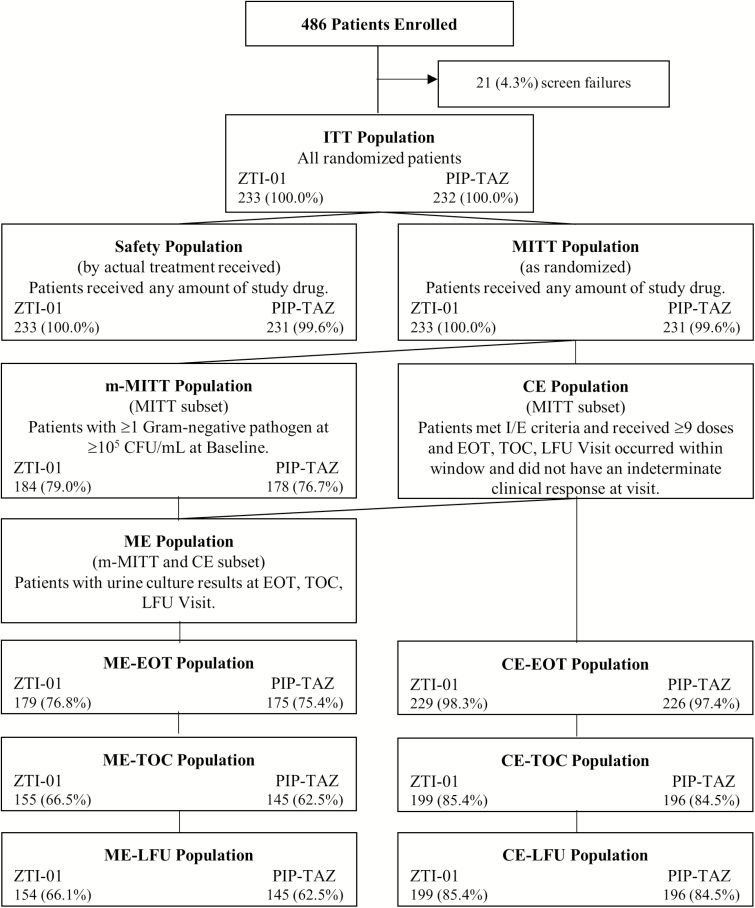
Analysis population disposition. Percentages were calculated using the number of patients in the ITT population as the denominator. Abbreviations: CE, clinical evaluable; CFU, colony-forming unit; EOT, end of treatment; I/E, inclusion/exclusion; ITT, intent-to-treat; LFU, late follow-up; ME, microbiologic evaluable; MITT, modified intent to treat; m-MITT, microbiologic modified intent to treat; PIP-TAZ, piperacillin-tazobactam; TOC, test of cure; ZTI-01, fosfomycin for injection.

Patients (N = 465) were included between May 2016 and January 2017; 464 patients received ≥1 dose of study drug (Figure 2). Patient demographics and baseline characteristics were well matched between treatment groups ( Table 1, Figure 2). Very few patients received prior antibiotics to treat their current cUTI/AP.

**Table 1.** Patient Demographics: Primary Analysis Population (Microbiologic Modified Intent-to-Treat)

	ZTI-01 (N = 184)	PIP-TAZ (N = 178)
Age, y, mean (SD)	49.9 (20.92)	51.3 (20.71)
Sex, n (%), Female:Male	119 (64.7):65 (35.3)	111 (62.4):67 (37.6)
Race		
White	184 (100)	178 (100)
BMI, kg/m^2^, mean (SD)	25.75 (5.26)	26.64 (5.84)
Primary diagnosis		
AP	100 (54.3)	96 (53.9)
cUTI	84 (45.7)	82 (46.1)
SIRS at baseline	62 (33.7)	52 (29.2)
Bacteremia at baseline	19 (10.3)	13 (7.3)
Estimated Charlson comorbidity index, mean (SD)	2.2 (2.63)	2.5 (2.93)
CrCl, mL/min, mean (SD)	83.6 (32.85)	84.7 (32.25)
CrCl, ≥20–50 mL/min	26 (14.1)	20 (11.2)
Baseline pathogen		
No prior short acting antibiotics	168 (91.3)	169 (94.9%)
Gram-negative Enterobacteriaceae	177 (96.2)	169 (94.9)
*Escherichia coli*	133 (72.3)	133 (74.7)
*Klebsiella pneumonia*	27 (14.7)	25 (14.0)
*Enterobacter cloacae* species complex	9 (4.9)	3 (1.7)
*Proteus mirabilis*	9 (4.9)	5 (2.8)
*Klebsiella oxytoca*	3 (1.6)	2 (1.1)
*Citrobacter amalonaticus/farmeri*	1 (0.5)	0
*Raoultella ornithinolytica*	1 (0.5)	1 (0.6)
*Serratia marcescens*	1 (0.5)	1 (0.6)
*Morganella morganii*	0	1 (0.6)
Gram-negative aerobes other than Enterobacteriaceae	10 (5.4)	9 (5.1)
*Pseudomonas aeruginosa*	8 (4.3)	9 (5.1)
*Acinetobacter baumannii-calcoaceticus* species complex	2 (1.1)	0
Gram-positive aerobes	4 (2.2)	8 (4.5)
*Enterococcus faecalis*	3 (1.6)	7 (3.9)
*Staphylococcus aureus*	1 (0.5)	0
*Staphylococcus saprophyticus*	0	1 (0.6)

The data represent n (%) unless otherwise specified.

Abbreviations: AP, acute pyelonephritis; BMI, body mass index; CrCl, creatinine clearance; cUTI, complicated urinary tract infection; PIP-TAZ, piperacillin-tazobactam; SD, standard deviation; SIRS, systemic inflammatory response syndrome; ZTI-01, fosfomycin for injection.

### Treatment

Median durations of IV therapy (safety population) were 7.1 days for both ZTI-01 and PIP-TAZ; 5/233 (2.1%) and 6/231 (2.6%) of patients in the ZTI-01 and PIP-TAZ groups, respectively, received 11–14 days of treatment for bacteremic cUTI/AP.

### Primary Efficacy

ZTI-01 was noninferior to PIP-TAZ for the primary efficacy outcome of overall success (clinical cure and microbiologic eradication) at TOC (m-MITT). Overall success occurred in 64.7% of ZTI-01 patients and 54.5% of PIP-TAZ patients (treatment difference 10.2%, 95% CI [−0.4, 20.8]) (Table 2).

**Table 2.** Overall, Clinical, and Microbiological Response by Analysis Populations

	Without PFGE (Uropathogen Identity Based on Species Name)			With PFGE (post hoc analysis) (Uropathogen I dentity Based on Molecular Typing)		
Population	ZTI-01, n (%)	PIP-TAZ, n (%)	Treatment Difference^a^ (95% CI)	ZTI-01, n (%)	PIP-TAZ, n (%)	Treatment Difference^a^ (95% CI)
TOC (m-MITT)						
Primary endpoint—overall response						
N	184	178	10.2 (−0.4, 20.8)	184	178	11.7 (1.3, 22.1)
Success	119 (64.7)	97 (54.5)		127 (69.0)	102 (57.3)	
Failure	54 (29.3)	73 (41.0)		46 (25.0)	68 (38.2)	
Indeterminate	11 (6.0)	8 (4.5)		11 (6.0)	8 (4.5)	
Secondary endpoint—clinical endpoint response						
Clinical response, N	184	178		NA	NA	NA
Cure	167 (90.8)	163 (91.6)	−0.8 (−7.2, 5.6)			
Failure	9 (4.9)	12 (6.7)				
Indeterminate	8 (4.3)	3 (1.7)				
Secondary endpoint—microbiological endpoint response						
N	184	178		184	178	
Eradication	121 (65.8)	100 (56.2)	9.6 (−1.0, 20.1)	130 (70.7)	107 (60.1)	10.5 (0.2, 20.8)
Persistence	50 (27.2)	69 (38.8)		41 (22.3)	62 (34.8)	
Indeterminate	13 (7.1)	9 (5.1)		13 (7.1)	9 (5.1)	
Overall response in patients with AP						
Overall response, N	99	94		99	94	
Success	67 (67.7)	62 (66.0)	1.7 (−12.6, 16.0)	71 (71.7)	62 (66.0)	5.8 (−8.3, 19.9)
Failure	25 (25.3)	29 (30.9)		21 (21.2)	29 (30.9)	
Indeterminate	7 (7.1)	3 (3.2)		7 (7.1)	3 (3.2)	
Overall response in patients with cUTI						
Overall response, N	85	84		85	84	
Success	52 (61.2)	35 (41.7)	19.5 (3.5, 35.5)	56 (65.9)	40 (47.6)	18.3 (2.4, 34.1)
Failure	29 (34.1)	44 (52.4)		25 (29.4)	39 (46.4)	
Indeterminate	4 (4.7)	5 (6.0)		4 (4.7)	5 (6.0)	
Overall response in patients with bacteremia at baseline						
Overall response, N	19	13		19	13	
Success	9 (47.4)	5 (38.5)	8.9 (−32.3, 50.1)	9 (47.4)	5 (38.5)	8.9 (−32.3, 50.1)
Failure	7 (36.8)	8 (61.5)		7 (36.8)	8 (61.5)	
Indeterminate	3 (15.8)	0 (0)		3 (15.8)	0 (0)	
Clinical response, N	19	13		NA	NA	NA
Cure	15 (78.9)	10 (76.9)	2.0 (−33.8, 37.8)			
Failure	2 (10.5)	3 (23.1)				
Indeterminate	2 (10.5)	0 (0)				
Microbiological response, N	19	13	1.2 (−40.5, 42.9)	NA	NA	NA
Eradication	9 (47.4)	6 (46.2)				
Persistence	6 (31.6)	7 (53.8)				
Indeterminate	4 (21.2)	0 (0)				
Estimated Charlson comorbidity index <3						
Overall response, N	115	107		NA	NA	NA
Success	85 (73.9)	68 (63.6)	10.4 (−2.7, 23.4)			
Failure	24 (20.9)	32 (29.9)				
Indeterminate	6 (5.2)	7 (6.5)				
Clinical response, N	115	107		NA	NA	NA
Cure	106 (92.2)	99 (92.5)	−0.3 (−8.2, 7.5)			
Failure	4 (3.5)	5 (4.7)				
Indeterminate	5 (4.3)	3 (2.8)				
Microbiological response, N	115	107	8.5 (−4.5, 21.5)	NA	NA	NA
Eradication	85 (73.9)	70 (65.4)				
Persistence	22 (19.1)	30 (28.0)				
Indeterminate	8 (7.0)	7 (6.5)				
Estimated Charlson comorbidity index ≥3						
Overall response, N	69	71		NA	NA	NA
Success	34 (49.3)	29 (40.8)	8.4 (−9.4, 26.3)			
Failure	30 (43.5)	41 (57.7)				
Indeterminate	5 (7.2)	1 (1.4)				
Clinical response, N	69	71		NA	NA	NA
Cure	61 (88.4)	64 (90.1)	−1.7 (−13.4, 9.9)			
Failure	5 (7.2)	7 (9.9)				
Indeterminate	3 (4.3)	0				
Microbiological response, N	69	71	9.9 (−8.0, 27.8)	NA	NA	NA
Eradication	36 (52.2)	30 (42.3)				
Persistence	28 (40.6)	39 (54.9)				
Indeterminate	5 (7.2)	2 (2.8)				
Prior antibiotic therapy category (no)						
Overall response, N	168	169		168		
Success	111 (66.1)	91 (53.8)	12.2 (1.3, 23.2)	117 (69.6)	95 (56.2)	13.4 (2.6, 24.2)
Failure	46 (27.4)	70 (41.4)		40 (23.8)	66 (39.1)	
Indeterminate	11 (6.5)	8 (4.7)		11 (6.5)	8 (4.7)	
Prior antibiotic therapy category (yes)						
Overall response, N	16	9		16	9	
Success	8 (50.0)	6 (66.7)	−16.7 (−64.7, 31.4)	10 (62.5)	7 (77.8)	−15.3 (−60.0, 29.5)
Failure	8 (50.0)	3 (33.3)		6 (37.5)	2 (22.2)	
LFU (m-MITT)						
Additional endpoint—clinical response at LFU						
Clinical response, N	184	178		NA	NA	NA
Sustained clinical cure	159 (86.4)	156 (87.6)	−1.2 (−8.7, 6.2)			
Relapse	8 (4.3)	7 (3.9)				
Clinical failure	9 (4.9)	12 (6.7)				
Indeterminate	8 (4.3)	3 (1.7)				
TOC (CE)						
Secondary endpoint—clinical endpoint response						
Clinical response, N	199	196		NA	NA	NA
Cure	188 (94.5)	182 (92.9)	1.6 (−3.7, 6.9)			
Failure	11 (5.5)	14 (7.1)				
TOC (ME)						
Secondary endpoint—clinical endpoint response						
Clinical response, N	155	145		NA	NA	NA
Cure	148 (95.5)	135 (93.1)	2.4 (−3.5, 8.3)			
Failure	7 (4.5)	10 (6.9)				

The following subgroup analyses were planned a priori: overall, clinical, and microbiological response responses by infection type (AP or cUTI), bacteremia, and prior antibiotic therapy. Subgroup analyses that were not predefined and performed post hoc included: responses by systemic inflammatory response syndrome criteria and estimates Charlson comorbidity index category at baseline. Estimated Charlson comorbidity index predicts 10-year survival in patients with multiple comorbidities and is based on medical history information obtained at baseline. Charlson comorbidity index was categorized as <3 and ≥3, and the numbers reported in this table are based on <3 category. N: Percentages are calculated using N, the number of patients in the corresponding analysis population as the denominator. PFGE was performed to molecularly type all baseline and TOC pathogens (both treatment arms), in order to confirm microbiological eradication/persistence; a total of 20 postbaseline pathogens were identified as unique, unrelated strains compared to baseline. The primary efficacy endpoint was the proportion of patients with an overall success (clinical cure and microbiologic eradication) in the m-MITT population at the TOC visit. Secondary efficacy endpoints included proportion of patients with a response of clinical cure in the MITT, m-MITT and CE, and ME populations at TOC, and proportion of patients with a response of microbiologic eradication in the m-MITT and ME populations at TOC.

Abbreviations: AP, acute pyelonephritis; CE, clinical evaluable; CI, confidence interval; cUTI, complicated urinary tract infection; LFU, late follow-up; ME, microbiologic evaluable; m-MMIT, microbiologic modified intent-to-treat; NA, not applicable; PFGE, pulsed-field gel electrophoresis; PIP-TAZ, piperacillin-tazobactam; TOC, test of cure; ZTI-01, fosfomycin for injection.

^a^Treatment difference was the difference in the clinical cure rate between the 2 treatment groups (ZTI-01 -PIP-TAZ). The 95% CIs (2-sided) were computed using a continuity-corrected Z-statistic.

Post hoc analysis of the primary endpoint using microbiological eradication rates based on PFGE molecular typing revealed an increase in overall success in both treatment arms. Treatment difference in overall success at TOC between treatment groups further increased in favor of ZTI-01 (treatment difference 11.7%, 95% CI [1.3, 22.1]) (Table 2).

### Secondary and Exploratory Efficacy Endpoints

Subgroup analyses of the primary endpoints (Table 2) were largely consistent across various baseline patient characteristics. Clinical cure rates were >90% in both treatment groups at TOC in MITT, m-MITT, CE, and ME groups (Table 2).

Baseline cUTI/AP caused by a baseline pathogen that was resistant to PIP-TAZ was uncommon (14 ZTI-01 patients, 9 PIP-TAZ patients). Of the 9 PIP-TAZ patients infected with a PIP-TAZ-resistant pathogen, 33.3% were clinical cures compared with 78.6% of ZTI-01 treated patients (TOC, Supplementary Appendix). Among patients infected with a PIP-TAZ-susceptible uropathogen at baseline, overall success rates were similar to those observed in the primary endpoint analysis (TOC, 64.7% ZTI-01, 56.7% PIP-TAZ).

### Microbiology

Most patients (92.4% ZTI-01, 94.4% PIP-TAZ, m-MITT) had a monomicrobial Gram-negative infection at baseline. Most common pathogens were *Escherichia coli* (72.3% ZTI-01, 74.7% PIP-TAZ) and *Klebsiella pneumoniae* (14.7% ZTI-01, 14.0% PIP-TAZ). All (100%) tested *E. coli* isolates in the ZTI-01 group were susceptible (provisional breakpoint MIC of ≤64 μg/mL), and 97.0% were susceptible to PIP-TAZ (MIC ≤16 μg/mL) (m-MITT).

Overall success (pathogens typed with PFGE analysis at TOC in m-MITT) occurred in 96/133 (72.2%) ZTI-01 and 77/133 (57.9%) PIP-TAZ patients who were infected with *E. coli* at baseline, and in 17/27 (63.0%) ZTI-01 and 13/25 (52.0%) PIP-TAZ patients who were infected at baseline with *K. pneumoniae*.

Clinical cure rates associated with *E. coli* and *K. pneumoniae* cUTI/AP were high and similar in both treatment groups (Table 3). Clinical cure rates in patients with severe disease (ie, met SIRS criteria or bacteremia) were high and similar between treatment groups (Table 2).

**Table 3.** Clinical and Microbiological Outcomes by Baseline Pathogen at Test of Cure (Microbiologic Modified Intent-to-Treat, Post Hoc Pulsed-field Gel Electrophoresis Analysis^a^)

Baseline Pathogen	Clinical Cure		Microbiologic Eradication	
	ZTI-01, n/N (%)	PIP-TAZ, n/N (%)	ZTI-01, n/N (%)	PIP-TAZ, n/N (%)
*Escherichia coli*	120/133 (90.2)	120/133 (90.2)	97/133 (72.9)	84/133 (63.2)
*Klebsiella pneumoniae*	25/27 (92.6)	25/25 (100)	18/27 (66.7)	14/25 (56.0)
*Proteus mirabilis*	8/9 (88.9)	3/5 (60.0)	8/9 (88.9)	1/5 (20.0)
*Enterobacter cloacae* species complex	8/9 (88.9)	3/3 (100)	6/9 (66.7)	3/3 (100)
*Klebsiella oxytoca*	2/3 (66.7)	2/2 (100)	2/3 (66.7)	2/2 (100)
*Raoultella ornithinolytica*	1/1 (100)	1/1 (100)	1/1 (100)	1/1 (100)
*Serratia marcescens*	1/1 (100)	1/1 (100)	1/1 (100)	0/1 (0)
*Morganella morganii*	0/0 (…)	1/1 (100)	0/0 (…)	1/1 (100)
*Citrobacter amalonaticus/farmer*	1/1 (100)	0/0 (…)	1/1 (100)	0/0 (…)
*Pseudomonas aeruginosa*	8/8 (100)	9/9 (100)	3/8 (37.5)	4/9 (44.4)
*Acinetobacter baumannii-calcoaceticus* species complex	2/2 (100)	0/0 (…)	2/2 (100)	0/0 (…)
*Enterococcus faecalis*	2/3 (66.7)	6/7 (85.7)	1/3 (33.3)	4/7 (57.1)
*Staphylococcus aureus*	1/1 (100)	0/0 (…)	1/1 (100)	0/0 (…)
*Staphylococcus saprophyticus*	0/0 (…)	1/1 (100)	0/0 (…)	1/1 (100)

Percentages were calculated using N as the denominator, where N was the number of patients with the specified pathogens. If both a urine and blood sample had the same pathogen at baseline, eradication was defined as the baseline bacterial pathogen was reduced to <10^4^ colony-forming unit/mL on urine culture and was negative on repeat blood culture.

Abbreviations: PIP-TAZ, piperacillin-tazobactam; ZTI-01, fosfomycin for injection.

^a^Pulsed-field gel electrophoresis was performed to molecularly type all baseline and test-of-cure pathogens (both treatment arms), in order to confirm microbiological eradication/persistence; a total of 20 postbaseline pathogens were identified as unique, unrelated strains compared to baseline.

Microbiologic eradication (PFGE-typed) occurred in 70.7% ZTI-01 versus 60.1% of PIP-TAZ patients (m-MITT) and 75.5% ZTI-01 versus 64.1% of PIP-TAZ patients (ME). Microbiological response rates varied between treatment groups among patients with severe disease; however, clinical cure rates in these subgroups were high and similar between treatment groups (TOC, Table 2).

Among patients with microbiological persistence at TOC, 14 patients (19.5% [8/41] ZTI-01, 9.7% [6/62] PIP-TAZ, TOC) had postbaseline growth of the baseline pathogen with ≥4-fold increase in MIC to study drug received; all had complicated disease, and 4 (3 ZTI-01, 1 PIP-TAZ) had recent or ongoing urinary tract instrumentation. A variety of pathogen species represented the isolates with ≥4-fold increase in MIC. Microbiological persistence rates associated with *Pseudomonas aeruginosa* were relatively high across the treatments (62.5% and 55.6%, respectively); however, each of these patients (8 ZTI-01, 9 PIP-TAZ patients) had explanations for *P. aeruginosa* persistence, including functional or anatomical abnormalities of the urinary tract (eg, atonic bladder, neurogenic bladder, hydronephrosis), indwelling instrumentation, and/or obstruction (eg, nephrolithiasis, stricture). Across either treatment, 100% of patients infected with *P. aeruginosa* were clinical cures (ie, requiring no further antibiotic), despite detection of *P. aeruginosa* persistence in postbaseline urine culture.

Overall, treatment arms were balanced in terms of number and type of baseline isolates bearing resistance characteristics (34%). Among these resistant isolates, clinical cure rates were high, and eradication rates numerically favored ZTI-01 (Table 4).

**Table 4.** Clinical and Microbiologic Outcomes Among Patients With Baseline Pathogens Demonstrating Phenotypic Resistance Characteristics (Test of Cure, Microbiologic Modified Intent-to-Treat) [9]

	ESBL		Amino-R		CRE		MDR	
	Cure, % (n/N)	Eradication, % (n/N)	Cure, % (n/N)	Eradication, % (n/N)	Cure, % (n/N)	Eradication, % (n/N)	Cure, % (n/N)	Eradication, % (n/N)
ZTI-01	93 (52/56)	55 (32/58)	97 (29/30)	67 (20/30)	100 (9/9)	56 (5/9)	92 (34/37)	54 (20/37)
PIP-TAZ	93 (51/55)	47 (27/57)	94 (29/31)	38 (12/32)	85 (11/13)	31 (4/13)	90 (28/31)	36 (12/33)

Using minimum inhibitory concentrations from an accompanying antibiotic panel or agar dilution supplemented with glucose 6-phosphate for fosfomycin, blood or urine isolates were identified to assess patient and microbiologic outcome. The following definitions were used for this assessment—ESBL: ≥2 µg/mL MIC for aztreonam, ceftazidime, or ceftriaxone; CRE: ≥4 µg/mL imipenem or meropenem; Amino-R: gentamicin ≥8 µg/mL or amikacin ≥32 µg/mL; MDR: nonsusceptibility ≥3 classes, using definitions above plus levofloxacin ≥4 µg/mL and trimethoprim/sulfamethoxazole ≥32 g/mL. Patients could have more than 1 isolate from blood and/or urine sources, and all organisms are presented for completeness. Patients with multiple organisms were counted only once per resistance grouping. If the same species was identified from a different source, the isolate was counted once for microbiological outcome.

Abbreviations: CRE, carbapenem-resistant Enterobacteriaceae; ESBL, extended-spectrum beta-lactamase; MDR, multidrug-resistant; MIC, minimum inhibitory concentration; ZTI-01, fosfomycin for injection.

### Safety

A total of 42.1% ZTI-01 patients and 32.0% PIP-TAZ patients experienced at least 1 TEAE (Table 5). Most were mild or moderate in severity; severe TEAEs were uncommon (2.1% ZTI-01, 1.7% PIP-TAZ). Most common TEAEs in both treatment groups were asymptomatic laboratory abnormalities and gastrointestinal events. Serious AEs (SAEs) were uncommon in both treatment groups (2.1% ZTI-01, 2.6% PIP-TAZ). There were no deaths in the study; 1 SAE in each treatment group was deemed related to study drug (hypokalemia in a ZTI-01 patient, renal impairment in a PIP-TAZ patient). Study drug discontinuations due to TEAEs were infrequent and similar between treatment groups (3.0% ZTI-01, 2.6% PIP-TAZ).

**Table 5.** Patients With Treatment-emergent Adverse Events (≥2% of Patients in Any Treatment Group) by System Organ Class and Preferred Term (Population: Safety)

System Organ Class Preferred Term	ZTI-01	PIP-TAZ
	(N = 233), n (%)	(N = 231), n (%)
Any AEs	99 (42.5)	74 (32.0)
Any TEAEs	98 (42.1)	74 (32.0)
Mild	84 (36.1)	49 (21.2)
Moderate	35 (15.0)	38 (16.5)
Severe	5 (2.1)	4 (1.7)
Drug-related TEAEs	48 (20.6)	32 (13.9)
SAEs	5 (2.1)	6 (2.6)
Drug-related SAE	1 (0.4)	1 (0.4)
TEAEs leading to study drug discontinuation	7 (3.0)	6 (2.6)
Serious TEAEs leading to study drug discontinuation	0 (0)	1 (0.4)
Gastrointestinal disorders	25 (10.7)	17 (7.4)
Nausea	10 (4.3)	3 (1.3)
Diarrhea	9 (3.9)	11 (4.8)
Vomiting	9 (3.9)	1 (0.4)
General disorders and administration site conditions	14 (6.0)	14 (6.1)
Infusion site phlebitis	2 (0.9)	6 (2.6)
Infections and infestations	17 (7.3)	20 (8.7)
Urinary tract infection	4 (1.7)	5 (2.2)
Investigations	20 (8.6)	8 (3.5)
Alanine aminotransferase increased	20 (8.6)	6 (2.6)
Aspartate aminotransferase increased	17 (7.3)	6 (2.6)
Metabolism and nutrition disorders	17 (7.3)	4 (1.7)
Hypokalemia	15 (6.4)	3 (1.3)
Nervous system disorders	10 (4.3)	7 (3.0)
Headache	6 (2.6)	5 (2.2)

Medical Dictionary for Regulatory Activities Version 19.0 was used to code TEAEs. Percentages were calculated using the number of patients in the safety population as the denominator. Treatment-emergent adverse events were defined as adverse events that newly appeared, increased in frequency, or worsened in severity following initiation of study drug. An adverse event was programmatically defined as treatment emergent if the start date and time was on or after the start date and time of the first dose of study drug.

Abbreviations: AE, adverse event; PIP-TAZ, piperacillin-tazobactam; SAE, serious adverse event; TEAE, treatment-emergent adverse event; fosfomycin for injection.

Most common laboratory abnormality TEAEs were increased ALT and AST (Table 5). None of the aminotransferase elevations were symptomatic or treatment-limiting. No patient met Hy’s law criteria. All cases exhibiting ALT or AST >3×ULN were adjudicated by an independent, blinded Transaminase Elevation Adjudication Committee; most ALT/AST elevations were deemed mild and possibly related to the study drug (23 ZTI-01, 5 PIP-TAZ cases).

Hypokalemia occurred in 71/232 (30.6%) ZTI-01 and 29/230 (12.6%) PIP-TAZ patients. Most cases were mild-to-moderate in severity; shifts in potassium levels from normal at baseline to hypokalemia were more frequent in the ZTI-01 group for mild (17.7% vs 11.3%), moderate (11.2% vs 0.9%), and severe (1.7% vs 0.4%) categories of hypokalemia. Hypokalemia was deemed a TEAE in 6.4% ZTI-01 and 1.3% PIP-TAZ, and all cases were transient and asymptomatic (Table 5). Although no significant cardiac AEs were observed, postbaseline QTcF of >450 to ≤480 msec (baseline QTcF of ≤450 msec) occurred at a higher frequency in ZTI-01 patients (7.3%) compared with PIP-TAZ (2.5%). In the ZTI-01 arm, these appear to correlate with the hypokalemia associated with IV formulation salt load. Only 1 patient (PIP-TAZ group) had a baseline QTcF of ≤500 msec and postbaseline QTcF of >500 msec.

## DISCUSSION

In ZEUS, ZTI-01 was noninferior to PIP-TAZ in overall success for treatment of cUTI/AP. Overall success occurred in 64.7% of ZTI-01 and 54.5% of PIP-TAZ patients (treatment difference of 10.2%, 95% CI [−0.4, 20.8]). Post hoc analysis of this primary endpoint using PFGE-typed uropathogens demonstrated an increased treatment difference favoring ZTI-01 (69.0% ZTI-01 vs 57.3% PIP-TAZ patients, treatment difference of 11.7% [95% CI: 1.3, 22.1]). Clinical cure rates were >90% in both treatment groups (Table 2). Microbiologic eradication rates at TOC (70.7% ZTI-01 vs 60.1% PIP-TAZ) were within range of those observed in contemporary cUTI trials, considering differences in timing of TOC, definitions of eradication, proportions of enrolled cUTI versus AP, instrumentation rates, and other factors [10, 11]. Most patients with microbiological persistence had identifiable reasons or risk factors for persistence. Notably, these patients were clinical cures at TOC, did not require rescue antimicrobial therapy, and remained sustained clinical cures at late follow-up. High dose of PIP-TAZ utilized in this study was according to current labeled guidelines for treating cUTI/AP [8], and the dosage was similar to that used in a recent registration cUTI/AP study [11].

Predominant uropathogens in both groups were *E. coli* and *K. pneumoniae* and are, in addition to other organisms recovered, representative of pathogens expected in US patients with cUTI/AP [12]. Clinical cure rates at TOC were high for ZTI-01 (>90%) for these 2 common pathogens, and microbiologic eradication rates were similar, 72.9% for *E. coli* and 66.7% for *K. pneumoniae*. Number of pathogens with ≥4-fold increase in MIC was low and similar between treatment groups. Main mechanism for the acquisition of fosfomycin resistance is impaired transport across the bacterial membrane, due to mutations of any of the target genes encoding antibiotic permeases (eg, glycerol-3-phosphate transporter and hexose-6-phosphate transporter in Enterobacteriaceae) [13]. Despite wide use of IV fosfomycin outside the United States for >40 years, resistance rates have remained low and stable [14]. Fosfomycin resistance appears to carry a biological fitness cost, which may in part explain the low prevalence of resistance and the paradox of high rates of clinical cure despite increased MIC [15].

Majority of TEAEs were mild or moderate in severity. The most common abnormal laboratory TEAEs were increased ALT, increased AST, and hypokalemia; these asymptomatic laboratory TEAEs occurred at higher frequencies in the ZTI-01 compared with the PIP-TAZ group, but none led to discontinuation of study drug. Aminotransferase elevation is a well-described AE of IV fosfomycin. In a recent review and analysis of the FDA AE reporting database (23 trials, N = 1242), the overall incidence of abnormal liver tests reported as AEs was approximately 1–2% among patients treated with various doses of IV fosfomycin (up to 24 g/day) [16]. No cases of severe hepatotoxicity leading to liver failure, liver transplant, or death have been reported with IV fosfomycin. An independent hepatologist committee determined that all cases with ALT or AST increases >3 × ULN were mild and asymptomatic, and no cases featured acute hypersensitivity, severe hepatotoxicity, or acute liver failure. In all cases, elevated aminotransferase values spontaneously returned to normal or baseline values after EOT. The clinical signature of ZTI-01-associated aminotransferase elevations is similar to that of other commonly used antibiotics.

Association of fosfomycin with abnormal electrolyte shifts (ie, hypokalemia and hypernatremia) is well described. Short fosfomycin infusions over 30 to 60 minutes, as were administered in this study, may be related to hypokalemia [17]. Imbalance of hypokalemia between the ZTI-01 and PIP-TAZ groups in this study may also be explained by the higher sodium load of the ZTI-01 formulation; each gram of ZTI-01 contains 330 mg of sodium.

### Limitations

The ZEUS study design may not reflect standard management of cUTI/AP in the United States because it was designed to reflect regulatory requirements. For example, a fixed regimen of 7 days of IV therapy with hospitalization was mandated (or longer for bacteremic patients), and no oral switch therapy or combination antimicrobial therapy was allowed. However, this study design allowed for frequent safety assessments, which better defined the laboratory abnormalities associated with this antibiotic (including ALT/AST and electrolyte values). Certain patient groups were excluded from the study, including patients with end-stage renal disease (eg, requiring dialysis), immunocompromised patients, and patients with monomicrobial Gram-positive cUTI/AP. A majority of patients were enrolled outside the United States where higher rates of antimicrobial resistance are observed.

## CONCLUSIONS

In this study, ZTI-01 met the primary objective of NI compared with PIP-TAZ in treatment of hospitalized patients with cUTI/AP. ZTI-01 provides a differentiated MOA with a broad spectrum of activity inclusive of Gram-negative pathogens, including ESBL producing and MDR Enterobacteriaceae. In the era of unmet medical needs due to growing concerns pertaining to MDR bacteria, ZTI-01 potentially offers an important novel IV therapeutic to the antibiotic armamentarium in the United States.

## Supplementary Data

Supplementary materials are available at *Clinical Infectious Diseases* online. Consisting of data provided by the authors to benefit the reader, the posted materials are not copyedited and are the sole responsibility of the authors, so questions or comments should be addressed to the corresponding author.

ciz181_suppl_Supplementary_Appendix

## Notes

***Acknowledgments.*** The authors thank the patients and their caregivers for their participation in this trial, the principal investigators, the clinical trial site staff members, trial coordinators, nursing and supporting staff, project managers, trial monitors, statisticians, remaining members of the trial team and Medical Practice (contract research organization), specifically Brian Murphy, MD, MPH, FIDSA, Vice President of Infectious Diseases and Medical Director at Medpace and Aníbal Calmaggi, MD, Sr. Medical Director Infectious Disease and Vaccines at Medpace. The authors thank the Data Monitoring Committee (Louis Rice, MD, Chair of the Department of Medicine at The Warren Alpert Medical School of Brown University and Chief of Medicine at Rhode Island Hospital and The Miriam Hospital; Keith Kaye, MD, University of Michigan; and Aaron Dane, Director at DaneStat Consulting, Cheshire, UK). The authors thank Donna Simcoe of Simcoe Consultants, Inc., funded by Zavante Therapeutics, Inc., San Diego, California, now a part of Nabriva Therapeutics plc, King of Prussia, Pennsylvania, for development of an early draft of the manuscript based on conversations with the authors. The authors express their sincere gratitude to the following principal investigators who participated in the trial: Igor Aboyan, MD/PhD, Zdorovye Clinical-diagnostic Center, Rostov, Russia; Salman Al-Shukri, MD/PhD/Professor, St. Petersburg State Medical University n.a. academician I.P. Pavlov, St. Petersburg, Russia; Andreeva, Alla, MD, Smolensk Regional Clinical Hospital, Smolensk, Russia; Antonian, Igor, MD, Kharkiv Medical Academy of Postgraduate Education, chair of General, Children and Oncology, Urology, based on Regional Clinical Center of Urology and Nephrology named after V. I. Shapoval, Kharkiv, Ukraine; Bako, Gabriel, MD/PhD, Spitalul Municipal Hospital “Dr. Gavril Curteanu,” Oradea, Nephrology, Romania; Banchev, Angel, MD/PhD, University Multiprofile Hospital for Active Treatment “Kaspela”; Urology Clinic, Plovdiv, Bulgaria; Bogov, Boris, MD, UMHAT Aleksandrovska—Clinic of Nephrology, Sofia, Bulgaria; Brodak, Milos, MD/PhD, Fakultni Nemocnice Hradec Kralove, Hradec Kralove, Czech Republic; Cabraja, Ivica, MD, General Hospital Dr. Josip Bencevic, Slavonski Brod, Croatia; Chkhotua, Archil, MD/PhD, Tsulukidze National Center of Urology, Tbilisi, Georgia; Chorvat, Erik, MD, Nemocnica Poprad, a.s. Urologicke oddeleni, Poprad, Slovakia; Didmanidze, Mzia, MD/PhD, Unimed Adjara LLC, Batumi, Georgia; Dosta, Nikolay, MD/PhD, Minsk Regional Clinical Hospital, Minsk, Belarus; Dudar, Iryna, MD/PhD, Efferent Technologies, Department of State Institution, Institute of Nephrology of NAMS of Ukraine, Kyiv, Ukraine; Dunets, Konstantin, MD, SPB GUZ City Hospital #15, St. Petersburg, Russia; Ephtimios, Issa, MD, Pensacola Research Consultants, Inc., Pensacola, FL, US; Falagas, Matthew, MD/PhD, IASO Hospital of Athens, Athens, Greece; Fedosiuk, Elena, MD, Brest Regional Hospital, Brest, Belarus; Fomkin, Roman, MD/PhD, Saratov State Medical University, Saratov, Russia; Galkina, Natalia Gennadieva, MD/PhD, Penza Regional Clinical Hospital n.a. Burdenko, Penza, Russia; Georgiev, Marin, MD/PhD, MHAT “Doverie,” Sofia, Bulgaria; Gratsianskaya, Anna Nikolaevna, MD/PhD, City Clinical Hospital #67, Moscow, Russia; Gumbrevicius, Gintautas, MD/PhD, Kauno Klinikine ligonine, Kaunas, Lithuania; Harris, Gehrig Jr., MD, Columbus Regional Research Institute, Columbus, Georgia, United States; Ismail, Gener, MD/PhD, Institutul Clinic Fundeni—Nephrology, Bucharest, Romania; Ivashchenko, Petro, MD, Kyiv City Clinical Hospital No.3; Urology Department, Kyiv, Ukraine; Kalachyk, Aleh, MD, City Clinical Hospital 9, Minsk, Belarus; Kameneva, Tatiana, MD/PhD, State Budget Healthcare Institution of Moscow City Hospital 3 of Moscow Healthcare Department, Moscow, Russia; Khodos, Valeriy, MD, Odesa City Clinical Hospital #10, Urology Department 1, Odesa, Ukraine; Kiknavelidze, Koba, MD/PhD, LTD Acad. Z. Tskhakaia West Georgian National Center of Interventional Medicine, Kutaisi, Georgia; Kivi, Martin, MD, East Tallinn Central Hospital, Tallinn, Estonia; Kliment, Jan, MD/PhD, Univerzitna nemocnica Martin Urologicka klinika, Martin, Slovakia; Kochiashvili, David, MD/PhD, Clinic Gid Medi, Tbilisi, Georgia; Kogan, Mikhail, MD/PhD, Rostov State Medical University, Rostov-On-Don, Russia; Kulchavenya, Ekaterina, MD, Novosibirsk Research Institute of Tuberculosis, Novosibirsk, Russia; Kurnatowska, Ilona, MD/PhD, Samodzielny Publiczny ZOZ Uniwersytecki Szpital Kliniczny nr. 1 im. Norberta Barlickiego Uniwersytetu Medycznego w Lodzi, Oddzial Kliniczny Nefrologii, Hipertensjologii i Transplantologii Nerek, Lodz, Poland; Laheaar, Marianne, MD, North Estonian Regional Hospital, Tallinn, Estonia; Lakatos, Akos, MD, Csongrad Megyei Dr. Bugyi Istvan Korhaz, Urologia, Szentes, Hungary; Lazanas, Marios, MD/PhD, Red Cross General Hospital; Department of Internal Medicine-Infectious Disease, Athens, Greece; Ledina, Dragan, MD/PhD, Clinical Hospital Centre Split, Split, Croatia; Lind, Jaak, MD, Ida-Viru Central Hospital, Kohtla Jarve, Estonia; Liptak, Jozsef, MD, Kanizsai Dorottya Korhaz; Urologiai Osztaly, Nagykanizsa, Hungary; Maciukiewicz, Piotr, MD/PhD, Szpital Specjalistyczny im. Ludwika Rydygiera w Krakowie, Oddzial Urologii, Krakow, Poland; Mangushlo, Alexander Alexandrovich, MD, St. Petersburg State Healthcare Institution—City Hospital 26, St. Petersburg, Russia; Marcisz, Czeslaw, MD/PhD, Megrez Sp. z o.o.; Oddzial Geriatryczny, Tychy, Poland; Marczewski, Krzysztof, MD/PhD, Samodzielny Publiczny Szpital Wojewodzki im. Papieza Jana Pawla II, Oddzial Nefrologii, Endokrynologii, Nadcisnienia Tetniczego i Chorob Wewnetrznych, Zamosc, Poland; Mariyanovski, Valeri, MD/PhD, Multiprofile Hospital for Active Teatment (MHAT) and Emergency Medicine—Pirogov, and MHAT and Emergency Medicine Clinic of Urology, Sofia, Bulgaria; Marusic, Srecko, MD, Clinical Hospital Dubrava, Zagreb, Croatia; Matevosyan, Tigran Rubenovich, MD, Vsevolozhsk Clinical Interdistrict Hospital, Vsevolozhsk, Russia; McCoy, Christopher, PharmD, Beth Israel Deaconess Medical Center, Boston, Massachusetts, United States; Mecl, Jan, MD, Krajska Nemocnice Liberec, Liberec, Czech Republic; Metallidis, Symeon, MD/PhD, AHEPA General University Hospital of Thessaloniki, Thessaloniki, Greece; Mickevicius, Ramunas, MD/PhD, Lietuvos sveikatos mokslu universiteto ligonine Kauno klinikos, Kaunas, Lithuania; Mikhailova_Mikhailava, Elena_Alena, MD, Gomel Regional Clinical Hospital, Gomel, Belarus; Mikulas, Juraj, MD/PhD, Fakultna nemocnica s poliklinikou Zilina, Zilina, Slovakia; Mitroi, George, MD/PhD, Spitalul Clinic Judetean de Urgenta Craiova, Craiova, Romania; Motylev, Igor, MD, City Hospital #33 of Leninsky district of Nizhny Novgorod, Nizhny Novgorod, Russia; Nadareishvilli, Akaki, MD/PhD, Research Institute of Clinical Medicine, Tbillisi, Georgia; Napora, Piotr,MD, Centrum Badan Klinicznych Piotr Napora Lekarze Sp. p., Wrocław, Poland; Pacik, Dalibor, MD/PhD, Fakultni nemocnice Brno, Brno, Czech Republic; Pap, Tibor, MD, CRU Hungary Kft., Miskolc, Hungary; Petersons, Alvars, MD/PhD, Paula Stradina kliniska universitates slimnica, Riga, Latvia; Pimanov, Sergey, MD/PhD, Vitebsk Regional Clinical Hospital, Vitebsk, Belarus; Pliszek, Krzysztof, MD, Szpital Wojewodzki w Bielsku Bialej; Szpital Wojewodzki Oddzial Urologii i Onkologii Urologiczne, Bielsko-Biala, Poland; Poirier, Robert, MD, Washington University School of Medicine, St. Louis, Missouri, United States; Popova, Veronika, MD, City Multifield Hospital 2, St. Petersburg, Russia; Puljiz, Ivan, MD/PhD, University Hospital for Infectious Diseases “Dr. Fran Mihaljevic” Department for general infectious diseases, Zagreb, Croatia; Pullman, John, MD, Mercury Street Medical, Butte, Montana, United States; Ramonas, Henrikas, MD/PhD, Vilniaus universitetine ligonine, Santariskiu Klinikos, Vilnius, Lithuania; Rossovskaya, Maria, MD/PhD, City Clinical Emergency Hospital n.a. N.S. Karpovich, Krasnoyarsk, Russia; Sagan, Olexiy, MD, PhD, Assistant Professor, Municipal Institution Zaporizhzhia Regional Clinical Hospital of Zaporizhzhia, Regional Council Department of Urology, State Institution Zaporizhzhia Medical Academy of Postgraduate Education under the Ministry of Health of Ukraine, Zaporizhzhia, Ukraine; Semenukha, Volodymyr, MD, Odesa Regional Clinical Hospital, Odesa, Ukraine; Shuliak, Alexander, MD, Clinic of State Institution “Institute of Urology of National Academy of Medical Sciences of Ukraine”—Department of Sexopathology and Andrology, Kyiv, Ukraine; Silinis, Darius, MD/PhD, Vilniaus miesto klinikine ligonine, Urologijos skyrius Vilnius, Lithuania; Sinelnikov, Lev, MD, Russian Military Medical Academy based at First Military Navy Hospital, St. Petersburg, Russia; Skerk, Visnja, MD/PhD, University Hospital for Infectious Diseases “Dr. Fran Mihaljevic” Department for urinary tract infections, Zagreb, Croatia; Skoutelis, Athanasios, MD/PhD, Evangelismos General Hospital of Athens, Athens, Greece; Spivak, Oleh, MD, Chernihiv City Hospital #2 of Chernihiv City Council, Department of Urology, Chernihiv, Ukraine; Stonkus, Sigitas, MD/PhD, Klaipedos universitetine ligonine, Vidaus ligu departamentas, Klaipeda, Lithuania; Stus, Viktor, MD/PhD, Municipal Institution Dnipropetrovsk Medical Academy of MOH of Ukraine, Dnipro, Ukraine; Sulowicz, Wladyslaw, MD/PhD, SCM Sp. z o.o., Krakow, Poland; Szabó, Attila, MD, Balassa Janos Korhaz, Szekszard, Hungary; Szalecki, Pawel, MD, HEUREKA Hanna Szalecka, Piaseczno, Poland; Szántó, Árpád, MD/PhD, PTE AOK Klinikai Kozpont, Urologiai Klinika, Pecs, Hungary; Tenke, Peter, MD, Jahn Ferenc Del-pesti Korhaz; Urology department, Budapest, Hungary; Teodorovich, Oleg, MD/PhD, Scientific Clinical Center of JSC Russian Railways, Moscow, Russia; Vazquez, Jose, MD, Augusta University (Georgia Regents University), Augusta, Georgia, United States; Vjaters, E, MD/PhD, Paula Stradina kliniska universitates slimnica, Urologijas centrs, Riga, Latvia; Vorps, Dainis, MD, Valmiera Slimnica, Valmiera, Latvia; Yakubtsevich, Ruslan, MD, Grodno Regional Clinical Hospital, Grodno, Belarus; Yanev, Krassimir, MD/PhD, UMHAT Aleksandrovska—Clinic of Urology, Clinic of Anaesthesiology and Intensive Care, Clinic of Clinical Hematology; Clinic of Urology, Sofia, Bulgaria; Zakharov, Konstantin, MD, Scientific Research Center Eco-Safety, St. Petersburg, Russia.

***Financial support***. This work was supported by Zavante Therapeutics, Inc., San Diego, California, now a part of Nabriva Therapeutics plc, King of Prussia, Pennsylvania.

***Potential conflicts of interest.*** L. B. R. and K. S. K. are members of the Data Monitoring Committee and consultants to Zavante Therapeutics, Inc., San Diego, California, now a part of Nabriva Therapeutics plc, King of Prussia, Pennsylvania. E. J. E.-G. and D. S. are employees and shareholders of Zavante Therapeutics, Inc., San Diego, California, now a part of Nabriva Therapeutics plc, King of Prussia, Pennsylvania. A. L. D., A. F. D., and P. B. E. are consultants to Zavante Therapeutics, Inc., San Diego, California, now a part of Nabriva Therapeutics plc, King of Prussia, Pennsylvania. A. L. D. reports personal fees from Zavante, Achaogen, AntibioTx, Cempra, Contrafect, IterumTx, Melinta, Nabriva, Paratek, Tetraphase, Theravance, Utility Therapeutics, and Wochhardt, outside the submitted work. P. B. E. reports personal fees from Spero Therapeutics, Utility Therapeutics, and Paratek Pharmaceuticals, outside the submitted work. K. S. K. reports personal fees from Zavante, Melinta, Allergan, Achaogen, Shinogi, and grants and personal fees from Merck, outside the submitted work. All authors have submitted the ICMJE Form for Disclosure of Potential Conflicts of Interest. Conflicts that the editors consider relevant to the content of the manuscript have been disclosed.

## References

[1] Alemayehu D, Quinn J, Cook J, Kunkel M, Knirsch CA A paradigm shift in drug development for treatment of rare multidrug-resistant gram-negative pathogens. Clin Infect Dis 2012; 55:562–7. 22610933 10.1093/cid/cis503

[2] Shields RK, Potoski BA, Haidar G, et al. Clinical outcomes, drug toxicity, and emergence of ceftazidime-avibactam resistance among patients treated for carbapenem-resistant Enterobacteriaceae infections. Clin Infect Dis 2016; 63:1615–8. 27624958 10.1093/cid/ciw636 PMC5146720

[3] Falagas ME, Vouloumanou EK, Samonis G, Vardakas KZ Fosfomycin. Clin Microbiol Rev 2016; 29:321–47. 26960938 10.1128/CMR.00068-15 PMC4786888

[4] Falagas ME, Maraki S, Karageorgopoulos DE, Kastoris AC, Mavromanolakis E, Samonis G Antimicrobial susceptibility of multidrug-resistant (MDR) and extensively drug-resistant (XDR) Enterobacteriaceae isolates to fosfomycin. Int J Antimicrob Agents 2010; 35:240–3. 20034765 10.1016/j.ijantimicag.2009.10.019

[5] Monurol (fosfomycin tromethamine) sachet prescribing information, 2011 February. Available at: http://www.accessdata.fda.gov/drugsatfda_docs/label/2008/050717s005lbl.pdf. Accessed 21 September 2017.

[6] Grabein B, Graninger W, Rodríguez Baño J, Dinh A, Liesenfeld DB Intravenous fosfomycin-back to the future: systematic review and meta-analysis of the clinical literature. Clin Microbiol Infect 2017; 23:363–72. 27956267 10.1016/j.cmi.2016.12.005

[7] World Medical Association. World Medical Association Declaration of Helsinki: ethical principles for medical research involving human subjects. Available at: http://www.wma.net/e/policy/b3.htm. Accessed 26 February 2019 2008. 10.1191/0969733002ne486xx 16010903

[8] Piperacillin/Tazobactam 4g/0.5g powder for solution for infusion. Available at: https://www.medicines.org.uk/emc/product/6526/smpc. Accessed 24 October 2018.

[9] Eckburg PB, Skarinsky D, Das A, Ellis-Grosse EJ. Phenotypic antibiotic resistance in ZEUS: multi-center, randomized, double-blind phase 2/3 study of ZTI-01 versus piperacillin-tazobactam (P-T) in the treatment of patients with complicated urinary tract infections (cUTI) including acute pyelonephritis (AP). ID Week. San Diego, California, 7 October 2017. Poster 1830.

[10] Wagenlehner FM, Sobel JD, Newell P, et al. Ceftazidime-avibactam versus doripenem for the treatment of complicated urinary tract infections, including acute pyelonephritis: RECAPTURE, a phase 3 randomized trial program. Clin Infect Dis 2016; 63:754–62. 27313268 10.1093/cid/ciw378 PMC4996135

[11] Kaye KS, Bhowmick T, Metallidis S, et al. Effect of meropenem-vaborbactam vs piperacillin-tazobactam on clinical cure or improvement and microbial eradication in complicated urinary tract infection: the TANGO I randomized clinical trial. JAMA 2018; 319:788–99. 29486041 10.1001/jama.2018.0438 PMC5838656

[12] Mocarski M, Zhao Q, Ding M, Dixit S, Lodise T 1041Economic burden associated with key Gram-negative pathogens among patients with complicated urinary tract infections across US hospitals. Open Forum Infect Dis 2014; 1(Suppl 1):S305.

[13] Castañeda-García A, Blázquez J, Rodríguez-Rojas A Molecular mechanisms and clinical impact of acquired and intrinsic fosfomycin resistance. Antibiotics (Basel) 2013; 2:217–36. 27029300 10.3390/antibiotics2020217 PMC4790336

[14] Karageorgopoulos DE, Wang R, Yu XH, Falagas ME Fosfomycin: evaluation of the published evidence on the emergence of antimicrobial resistance in Gram-negative pathogens. J Antimicrob Chemother 2012; 67:255–68. 22096042 10.1093/jac/dkr466

[15] Pourbaix A, Guérin F, Lastours V, et al. Biological cost of fosfomycin resistance in *Escherichia coli* in a murine model of urinary tract infection. Int J Med Microbiol 2017; 307:452–9. 28986014 10.1016/j.ijmm.2017.09.019

[16] Iarikov D, Wassel R, Farley J, Nambiar S Adverse events associated with fosfomycin use: review of the literature and analyses of the FDA adverse event reporting system database. Infect Dis Ther 2015; 4:433–58. 26437630 10.1007/s40121-015-0092-8 PMC4675770

[17] Florent A, Chichmanian RM, Cua E, Pulcini C Adverse events associated with intravenous fosfomycin. Int J Antimicrob Agents 2011; 37:82–3. 21074377 10.1016/j.ijantimicag.2010.09.002

