# Supplementary material for: Fosfomycin for Injection (ZTI-01) Versus Piperacillin-tazobactam for the Treatment of Complicated Urinary Tract Infection Including Acute Pyelonephritis: ZEUS, A Phase 2/3 Randomized Trial
Source: Clin Infect Dis. 2019 Mar 6;69(12):2045–56. doi: 10.1093/cid/ciz181 (PMC6880332; doi:10.1093/cid/ciz181)
Supplement: ciz181_suppl_Supplementary_Appendix [file ciz181_suppl_supplementary_appendix.docx]

# Supplementary Data

**­ZTI-01 (fosfomycin for injection) vs Piperacillin-Tazobactam for the Treatment of Complicated Urinary Tract Infection (cUTI) Including Acute Pyelonephritis (AP): ZEUS, A Phase 2/3 Randomized Trial**

Keith S. Kaye, MD, MPH,^1^ Louis B. Rice, MD,^2^ Aaron Dane, MSc,^3^ Viktor Stus, MD, PhD,^4^ Olexiy Sagan, MD,^5^ Elena Fedosiuk, MD,^6^ Anita Das, PhD,^7^ David Skarinsky, BS,^8^ Paul B Eckburg, MD,^9^ Evelyn J. Ellis-Grosse, PhD^10^

^1^Director of Clinical Research, Division of Infectious Diseases, University of Michigan Medical School, Ann Arbor, MI, US, [keithka@med.umich.edu](mailto:keithka@med.umich.edu)

^2^Chair of the Department of Medicine at The Warren Alpert Medical School of Brown University and Chief of Medicine at Rhode Island Hospital and The Miriam Hospital, Providence, RI, US, [LRICE@lifespan.org](mailto:LRICE@lifespan.org)

^3^Director, DaneStat Consulting, Alderly Edge, UK, [aarondane@danestat.com](mailto:aarondane@danestat.com)

^4^Doctor of Medical Science, Professor, Municipal Institution Dnipropetrovsk Medical Academy of MOH of Ukraine, Dnipro, Ukraine, [viktor.stus@gmail.com](mailto:viktor.stus@gmail.com)

^5^Assistant Professor, Municipal Institution Zaporizhzhia Regional Clinical Hospital of Zaporizhzhia, Regional Council Department of Urology, State Institution Zaporizhzhia Medical Academy of Postgraduate Education under the Ministry of Health of Ukraine, [olexiysagan@yahoo.com](mailto:olexiysagan@yahoo.com)

^6^Brest Regional Hospital, Brest, Belarus, [lenafedosyuk@mail.ru](mailto:lenafedosyuk@mail.ru)

^7^Das Statistical Consulting, Guerneville, CA, [Adas@ADstat.onmicrosoft.com](mailto:Adas@ADstat.onmicrosoft.com)

^8^VP of Clinical Operations, Zavante Therapeutics, Inc., San Diego, CA, now a part of Nabriva Therapeutics plc, King of Prussia, PA, US, [David.Skarinsky@Nabriva.com](mailto:David.Skarinsky@Nabriva.com)

^9^Acting Chief Medical Officer, Zavante Therapeutics, Inc., San Diego, CA, now a part of Nabriva Therapeutics plc, King of Prussia, PA, US, [Paul.Eckburg@Nabriva.com](mailto:Paul.Eckburg@Nabriva.com)

^10^Chief Scientific Officer, Zavante Therapeutics, Inc., San Diego, CA, now a part of Nabriva Therapeutics plc, King of Prussia, PA, US, [Evelyn.Ellis.Grosse@Nabriva.com](mailto:Evelyn.Ellis.Grosse@Nabriva.com)

**Inclusion Criteria**

Patients who meet all of the following criteria were eligible to participate in the study:

1. A signed informed consent form or, in case of a lack of decision-making capacity and as permitted by local law and institutional Standard Operating Procedures, consent on behalf of study patient by a legally authorized representative;
2. Male or female, ≥18 years of age;
3. Expectation, in the judgment of the Investigator, that the patient’s cUTI or AP would require hospitalization and treatment with IV antibiotics;
4. Documented or suspected cUTI or AP as defined below:
5. **cUTI:**

- Signs or symptoms evidenced by ≥2 of the following:
- Chills, rigors, or warmth associated with fever;

Note: Fever must have been observed and documented by a health care provider within 24 hours of Screening (oral, tympanic, rectal, or core temperature >38°C [>100.4°F]);

- Nausea or vomiting within 24 hours of Screening, as reported by the patient;
- Dysuria, increased urinary frequency, or urinary urgency;
- Lower abdominal pain or pelvic pain;
- *And* urine specimen with evidence of pyuria:
- Positive leukocyte esterase on urinalysis; or
- White blood cell count (WBC) ≥10 cells/mm^3^ in unspun urine; or
- WBC count ≥10 cells/high-power field (hpf) in urine sediment;
- *And* ≥1 of the following associated risks:
- Use of intermittent bladder catheterization or presence of an indwelling bladder catheter (Note: indwelling bladder catheters that had been in place for >24 hours prior to Screening must have been removed or replaced prior to collection of the Screening urine for urinalysis and culture, unless removal or replacement was considered unsafe or contraindicated);
- Current known functional or anatomical abnormality of the urogenital tract, including anatomic malformations or neurogenic bladder, or with a post-void residual urine volume of ≥100 mL;
- Complete or partial obstructive uropathy (eg, nephrolithiasis, tumor, fibrosis, urethral stricture) that was expected to be medically or surgically treated during study drug therapy (prior to EOT);
- Azotemia, defined as blood urea nitrogen (BUN) >20 mg/dL, blood urea >42.8 mg/dL, or serum creatinine >1.4 mg/dL, due to known prior intrinsic renal disease;
- Chronic urinary retention in men, for example, previously diagnosed benign prostatic hypertrophy;

1. **AP:**

- Signs or symptoms evidenced by ≥2 of the following:
- Chills, rigors, or warmth associated with fever;

Note: Fever must have been observed and documented by a health care provider within 24 hours of Screening (oral, tympanic, rectal, or core temperature >38°C [>100.4°F]);

- Nausea or vomiting within 24 hours of Screening, as reported by the patient;
- Dysuria, increased urinary frequency, or urinary urgency;
- Acute flank pain (onset within 7 days prior to randomization) or costo­vertebral angle tenderness on physical examination;
- *And* urine specimen with evidence of pyuria:
- Positive leukocyte esterase on urinalysis; or
- WBC count ≥10 cells/mm^3^ in unspun urine; or
- WBC count ≥10 cells/hpf in urine sediment;

1. Had a baseline urine culture specimen obtained within 48 hours prior to randomization;

Note: Patients may have been randomized into this study and started IV study drug therapy before the Investigator knew the results of the baseline urine culture;

1. Expectation, in the judgment of the Investigator, that any implanted urinary instrumentation (eg, nephrostomy tubes, ureteric stents) were to be surgically removed or replaced before or within 24 hours after randomization, unless removal or replacement was considered unsafe or contraindicated;

Note: Temporary bladder catheters that had been in place for >24 hours prior to Screening must have been removed or replaced prior to collection of the Screening urine for urinalysis and culture, unless removal or replacement was considered unsafe or contraindicated;

1. Expectation, in the judgement of the Investigator, that the patient would survive with effective antibiotic therapy and appropriate supportive care for the anticipated duration of the study;
2. The patient required initial hospitalization to manage the cUTI or AP in accordance with the standard of care;
3. Women of childbearing potential (ie, not post-menopausal or surgically sterilized) must have had a negative pregnancy test before randomization. Participating women of childbearing potential, or partners of participants who were of childbearing potential, must have been willing to consistently use a highly effective method of contraception between Screening and the end of the study (LFU Visit);

Note: Highly effective methods of contraception included the following: hormonal implants/patches, injectable hormones, oral hormonal contraceptives, intra-uterine devices, approved cervical rings, prior bilateral oophorectomy, prior hysterectomy, prior bilateral tubal ligation, true abstinence (if approved by the Investigator), or a vasectomized partner;

1. Male study participants were required to use condoms with a spermicide during sexual intercourse from Screening to the end of the study, even if their sexual partner was or may have been pregnant.

**Exclusion Criteria**

Patients who meet any of the following criteria will be excluded from participation in the study:

1. Presence of any known or suspected disease or condition that, in the opinion of the Investigator, may have confounded the assessment of efficacy, including, but not limited to, the following:
2. Perinephric abscess;
3. Renal corticomedullary abscess;
4. uUTI;
5. Any recent history of trauma to the pelvis or urinary tract;
6. Polycystic kidney disease;
7. Chronic vesicoureteral reflux;
8. Previous or planned renal transplantation;
9. Patients receiving dialysis, including hemodialysis, peritoneal dialysis, or continuous venovenous hemofiltration;
10. Previous or planned cystectomy or ileal loop surgery;
11. Known or suspected infection that was caused by pathogen(s) that was resistant to either study drug (fosfomycin or a BL/BLI combination), including infection caused by fungi (eg, candiduria) or mycobacteria (eg, urogenital tuberculosis);
12. Presence of suspected or confirmed acute bacterial prostatitis, orchitis, epididymitis, or chronic bacterial prostatitis as determined by history and/or physical examination;
13. Gross hematuria requiring intervention other than administration of study drug or removal or exchange of a urinary catheter;
14. Urinary tract surgery within 7 days prior to randomization or urinary tract surgery planned during the study period (except surgery required to relieve an obstruction or place a stent or nephrostomy prior to EOT);
15. Renal function at Screening as estimated by CrCl <20 mL/min using the Cockcroft-Gault formula and serum creatinine value obtained from a local laboratory;
16. Known non-renal source of infection such as endocarditis, osteomyelitis, abscess, meningitis, or pneumonia diagnosed within 7 days prior to randomization;
17. Any signs of severe sepsis, including but not limited to the following:
18. Shock or profound hypotension defined as systolic blood pressure <90 mmHg or a decrease of >40 mmHg from baseline (if known) that was not responsive to fluid challenge;
19. Disseminated intravascular coagulation as evidenced by prothrombin time or partial thromboplastin time ≥2 × the upper limit of normal (ULN) or <50,000 platelets/mm^3^ at Screening in patients in whom severe sepsis was suspected;
20. Pregnant or breastfeeding women;
21. Known seizure disorder requiring current treatment with anti-seizure medication which, in the Investigator’s opinion, would have prohibited the patient from complying with the protocol. Patients with a history of epilepsy or who were on stable treatment (ie, no change in therapy within 30 days) with well-controlled seizure disorder (ie, no recurrent episodes in past 30 days) may have been considered for enrollment in the study;
22. Treatment within 30 days prior to randomization with any cancer chemotherapy, immunosuppressive medications for transplantation, or medications for rejection of transplantation;
23. Evidence of significant hepatic disease or dysfunction, including known acute viral hepatitis or hepatic encephalopathy;
24. Aspartate aminotransferase (AST) or alanine aminotransferase (ALT) >5 × ULN or total bilirubin >3 × ULN at Screening;
25. Receipt of any potentially effective systemic antibiotic with activity against Gram­negative uropathogens for more than 24 hours within the 72-hour window prior to randomization. However, patients may have enrolled who:
26. Had received >48 hours of prior antimicrobial therapy and, (1) in the Investigator’s opinion, failed that preceding antimicrobial therapy (ie, had worsening signs and symptoms), and (2) were documented to have had a cUTI or AP that was caused by a pathogen resistant to the prior therapy, and (3) the causative pathogen was known not to be resistant (eg, the causative pathogen was either susceptible, intermediate, or unknown susceptibility) to fosfomycin or a BL/BLI combination;
27. Developed signs and symptoms of cUTI or AP while taking a systemic antibiotic for another indication (other than fosfomycin or BL/BLI combination), including antimicrobial prophylaxis for recurrent UTI;
28. Received a single dose of a short-acting (ie, having a dosage frequency of more than once daily [eg, every 12 hours or more frequently]) systemic antibiotic up to 24 hours prior to randomization (see Appendix 16.1.1 Protocol Amendment 2, Version 3.0, Appendix C – Short-Acting Antibiotics for definition and a list of allowed short‑acting antibiotics). No more than 25% of patients were enrolled who met this criterion;
29. Requirement at time of randomization for any reason for additional systemic antimicrobial therapy (including antibacterial, antimycobacterial, or antifungal therapy) other than study drug, with the exception of a single oral dose of any antifungal treatment for vaginal candidiasis;
30. Likely to require the use of an antibiotic for cUTI or AP prophylaxis during the patient’s participation in the study (from randomization through the LFU Visit);
31. Known history of human immunodeficiency virus infection and known recent CD4 count <200/mm^3^ within the last year;
32. Presence of significant immunodeficiency or an immunocompromised condition, including hematologic malignancy, bone marrow transplant, or receipt of immunosuppressive therapy such as cancer chemotherapy, medications for the rejection of transplantation, and long-term use of systemic corticosteroids (equivalent to ≥20 mg/day of prednisone or systemic equivalent for ≥2 weeks);
33. Presence of neutropenia (absolute neutrophil count <1,000/mm^3^) obtained at local laboratory at Screening;
34. Presence of thrombocytopenia (especially in patients diagnosed with disseminated intravascular coagulation or at risk of serious bleeding) <50,000 platelets/mm^3^ obtained at local laboratory at Screening;
35. A QT interval corrected using Fridericia’s formula (QTcF) >480 msec;
36. History of significant hypersensitivity or allergic reaction to fosfomycin, any contraindication to the use of piperacillin-tazobactam (PIP-TAZ) based on local approved prescribing information (eg, Summary of Medicinal Product Characteristics [SmPC]), any contraindication to the excipients used in the respective formulations, or any contraindication to the use of β‑lactam antibiotics (eg, cephalosporins, penicillins, carbapenems, or monobactams);
37. Participation in a clinical study involving investigational medication or investigational device within the last 30 days prior to randomization;
38. Inability, in the judgment of the Investigator, to tolerate the salt load required for study drug administration;
39. Unable or unwilling, in the judgment of the Investigator, to comply with the protocol;
40. Any patients previously randomized in this study.

**Dose Adjustments for Patients with Moderate or Severe Renal Impairment**

Azotemia was defined as blood urea nitrogen (BUN) >20 mg/dL, blood urea >42.8 mg/dL, or serum creatinine > 1.4 mg/dL, due to known prior intrinsic renal disease.

**ZTI-01**

Patients’ renal function was monitored by obtaining serum creatinine from the local laboratory, especially in patients with a known or suspected renal insufficiency. Estimated CrCl was calculated using the Cockcroft-Gault formula every time a local laboratory assessment of serum creatinine was performed. Actual weight in kilograms was required for the calculation and was obtained at Baseline and during all complete physical examinations throughout the study. The baseline Day 1 actual weight may have been used throughout the study in the Cockcroft-Gault formula to calculate CrCl if repeated weights could not be obtained.

Patients with a CrCl <20 mL/min at Screening based on local laboratory serum creatinine were excluded from study participation (see Exclusion Criterion 5). After randomization during the treatment period, study drug was discontinued for all patients with an estimated CrCl <20 mL/min.

Dose adjustment based on CrCl was only required for administration of ZTI-01 in patients with a CrCl ≤50 mL/min. Since the study was blinded, local laboratory serum creatinine was evaluated for all patients within 24 hours of the first dose of study drug to estimate CrCl and a potential for dose adjustment in the ZTI-01 treatment group. Patients with CrCl ≤50 mL/min at screening had a local serum creatinine collected to assess CrCl at least once daily (or more frequently) up to EOT, to ensure that ZTI-01 dosage was adjusted as necessary.

Table 1 displays the recommended dosage regimen adjustment for ZTI-01 in patients with CrCl ≥20 mL/min and ≤50 mL/min.

Supplementary Appendix Table 1. Recommended Dosage Regimen for ZTI-01 in Patients with Creatinine Clearance ≥20 mL/min and ≤50 mL/min

| CrCl (mL/min) | Treatment |
| --- | --- |
| >50 | 6 g ZTI-01 q8h (no adjustment) |
| >40 to ≤50 | 4 g ZTI-01 q8h |
| >30 to ≤40 | 6 g ZTI-01 once, then 3 g ZTI-01 q8h  (the 3 g will be administered 8 hours after the 6 g dose) |
| ≥20 to ≤30 | 6 g ZTI-01 once, then 5 g ZTI-01 q24h  (the 5 g will be administered 24 hours after the 6 g dose) |
| ZTI-01 was administered as a 200 mL infusion over 1 hour.  CrCl = creatinine clearance; q8h = every 8 hours; q24h = every 24 hours.  Source: Study Protocol (Appendix 16.1.1) | |

To maintain the blind and maintain q8h dosing when ZTI-01 was not being administered in patients with renal insufficiency requiring dose adjustment, “dummy” infusions of WFI were administered that contained no active ZTI-01.

**PIP-TAZ**

No dose adjustment was required for PIP-TAZ for patients with CrCl ≤20 mL/min, and patients received the 4.5 g dose q8h in WFI, as recommended by labeling (eg, PIP-TAZ SmPC).

**Schedule of Procedures - 7-Day Treatment Period**

| Procedure | Screening | Treatment | | | | | | EOT | TOC | LFU |
| --- | --- | --- | --- | --- | --- | --- | --- | --- | --- | --- |
|  | -48 hrs prior to D 1 [a] | Day 1  [b] | Day 2 [c] | Day 3 [c] | Day 4 [c] | Day 5 [c] | Day 6 [c] | Day 7+1 d or final dosing day +1 d | Day 19 +2 d  [c, d] | Day 26 ±2 d [d] |
| Informed consent | X |  |  |  |  |  |  |  |  |  |
| Inclusion/exclusion criteria | X |  |  |  |  |  |  |  |  |  |
| Medical/surgical history | X [e] |  |  |  |  |  |  |  |  |  |
| Prior/concomitant medications | X [f] |  | X | X | X | X | X | X | X | X |
| Demographics [g] | X |  |  |  |  |  |  |  |  |  |
| Complete physical examination [h] | X |  |  | X |  | X |  | X |  |  |
| Limited physical examination [h] |  |  | X |  | X |  | X |  | X | X |
| Vital signs [i] | X | X | X | X | X | X | X | X | X | X |
| Assess clinical signs/symptoms | X |  | X | X | X | X | X | X | X | X |
| Assess clinical outcome [j] |  |  |  |  |  | X |  | X | X [j] | X [j] |
| Randomization | X |  |  |  |  |  |  |  |  |  |
| Creatinine clearance [k] | X | X | X | X | X | X | X | X |  |  |
| Pregnancy test [l] | X | X |  |  |  |  |  | X |  |  |
| Serum chemistry [m] | X [n] | X | X | X | X | X | X | X | X | X |
| Hematology [o] | X [n] | X |  | X |  | X |  | X | X | X |
| Urinalysis [p] | X [n] | X |  | X |  | X |  | X | X | X |
| 12-lead ECG | X [q] |  |  | X [q] | | |  |  |  |  |
| Blood cultures | X | | X [r] | X [r] | X [r] | X [r] | X [r] | X [r] | X [r] | X [r] |
| Urine cultures | X [s] | |  |  |  | X [s] |  | X [s] | X [s] | X [s] |
| Administer study drug |  | X | X | X | X | X | X | X |  |  |
| Assessment of adverse events | X | X | X | X | X | X | X | X | X | X |
| PK samples |  | X [t] |  | X [t] | | |  |  |  |  |

1. Screening procedures must have been completed prior to randomization on Day 1. Screening laboratories for eligibility assessment were performed at the local laboratory and may have been collected as standard of care up to 48 hours prior to randomization.
2. Baseline (Day 1 pre-dose) chemistry, hematology, and urinalysis samples detailed in Appendix B of the Study Protocol must have been collected prior to the first dose of study drug and sent to the central laboratory. Urine samples taken 48 hours prior to randomization could have been used for baseline microbiology assessment if the organism(s) cultured were sent to the designated central laboratory for baseline. Otherwise, a repeat urine sample for baseline microbiologic assessment was required.
3. Study procedures were only required on treatment days that the patient received IV therapy. If EOT occurred before Day 7, study procedures were not required to be performed on subsequent days until the TOC Visit.
4. The TOC Visit occurred on Day 19 (+2 days). The LFU Visit occurred on Day 26 (±2 days). The LFU Visit was performed as an in-office visit; however, if the patient was unable to attend the LFU Visit in person, then the patient was contacted by telephone call for follow-up assessment of concomitant medications, clinical signs and symptoms, and adverse events.
5. Obtained medical/surgical history, including urological history; recorded inactive conditions diagnosed within the previous 5 years, completed urological/renal history, and all active conditions.
6. Reasonable effort was made to determine all relevant treatments (including all antibiotics, prescription and non-prescription medications, herbal medications, and vitamin supplements, supportive therapies, and non-pharmacologic treatments) received within 14 days before randomization and during the study.
7. Demographic data were collected, including name, sex, gender, race, and ethnicity.
8. Complete physical examination included weight (and height at Screening only) plus source documentation of skin, head and neck, heart, lung, abdomen, extremities, back/flank/costo-vertebral angle tenderness, and neuromuscular assessments. A Limited physical examination did not include weight and was a symptom based assessment.
9. Pre-dose vital signs included blood pressure, heart rate, respiratory rate, and temperature. Maximum daily temperature (defined as the maximum temperature reported on a single calendar day) was recorded. Body temperature was taken per the site’s preferred method but limited to oral, tympanic, rectal, or core measurements. The same method of measuring a patient’s body temperature was used throughout the study.
10. If a patient was a clinical failure at EOT, the patient was automatically considered a failure at the TOC and LFU visits; the assessment of clinical response by the Investigator was listed as “failure at EOT.”
11. Dose adjustment based on CrCl was only required for administration of ZTI-01 in patients with a CrCl ≤50 mL/min. At any time between Screening and EOT, local serum creatinine was obtained for CrCl determination at least once daily, or more frequently as needed.
12. For women of childbearing potential, a urine or serum pregnancy test was performed at Screening locally; a negative result within 24 hours of randomization was sufficient for eligibility assessment. A serum pregnancy test was performed by the central laboratory on Day 1 pre-dose; however, results were not required prior to dosing. At EOT, a local laboratory urine or serum pregnancy test was repeated for women of childbearing potential.
13. See Appendix B of the Study Protocol for a full list of serum chemistry panel reported at the central laboratory.
14. Collected Screening local lab samples for assessments of eligibility included: serum creatinine (for CrCl), PT/PTT, platelet count, ALT, AST, total bilirubin, absolute neutrophil count, blood urea nitrogen, blood urea, and urinalysis with microscopy (see Appendix B of the Study Protocol).
15. See Appendix B of the Study Protocol for a full list of hematology panel reported at the central laboratory.
16. See Appendix B of the Study Protocol for a full list of urinalysis panel and microscopy reported at the central laboratory.
17. The 12-lead ECG was performed at Screening and within 13 hours after the start of study drug administration on 1 of the days at which the post­dose PK samples were drawn (either Day 3, Day 4, or Day 5). The day on which the post-dose PK samples were drawn was at the discretion of the Investigator. All 12-lead ECGs were performed after the patient had been in a supine position for ≥10 min.
18. Repeat blood cultures were collected on the day that the positive blood culture was detected. If subsequent blood cultures were also positive, the blood cultures were repeated as necessary until negative blood cultures were obtained.
19. An adequate clean-catch urine specimen for culture (or other appropriate method to collect a urine culture that minimized risk of bacterial contamination) was obtained at the specified time points. At any point in the study, if a patient failed while on therapy, a urine specimen was obtained.
20. PK samples were collected immediately before the first dose of study drug and again 1, 2, 4, and 8 hours after study drug administration on 1 of the following days: either on Day 3, Day 4, or Day 5. The day on which the post-dose PK samples were drawn was at the discretion of the Investigator.

ALT = alanine aminotransferase; AST = aspartate aminotransferase; CrCl = creatinine clearance; ECG = electrocardiogram; EOT = End-of-Treatment; IV = intravenous; LDH = lactate dehydrogenase; LFU = Late Follow-up; PK = pharmacokinetic; PT = prothrombin time; PTT = partial thromboplastin time; q8h = every 8 hours; TOC = Test-of-Cure.

**Schedule of Procedures - Extension Up to 14 Days of Treatment**

| Procedure | Screening | Treatment | | EOT | TOC | LFU |
| --- | --- | --- | --- | --- | --- | --- |
|  |  | Days 1-7 | Days 8-13 [c] | Day 14+1 d or final dosing day +1 d | Day 19 +2  d  [c, d] | Day 26 ±2 d  [d] |
| Informed consent | See Schedule of Procedures Table 5: timing of procedures Screening to Day 7 Visit remain the same | |  |  |  |  |
| Inclusion/exclusion criteria |  |  |  |  |  |  |
| Medical/surgical history |  |  |  |  |  |  |
| Prior/concomitant medications |  |  | X | X | X | X |
| Complete physical examination [h] |  |  |  | X |  |  |
| Limited physical examination [h] |  |  | X |  | X | X |
| Vital signs [i] |  |  | X | X | X | X |
| Assess clinical signs/symptoms |  |  | X | X | X | X |
| Assess clinical outcome [j] |  |  |  | X | X [j] | X [j] |
| Randomization |  |  |  |  |  |  |
| Creatinine clearance [k] |  |  | X | X |  |  |
| Pregnancy test [l] |  |  |  | X |  |  |
| Serum chemistry [m] |  |  |  | X | X | X |
| Hematology [o] |  |  |  | X | X | X |
| Urinalysis [p] |  |  |  | X | X | X |
| 12-lead ECG |  |  |  |  |  |  |
| Blood cultures |  |  | X [r] | X [r] | X [r] | X [r] |
| Urine cultures |  |  |  | X [s] | X [s] | X [s] |
| Administer study drug |  |  | X | X |  |  |
| Assessment of adverse events |  |  | X | X | X | X |

ECG = electrocardiogram; EOT = End-of-Treatment; IV = intravenous; LFU = Late Follow-up; PK = pharmacokinetic; TOC = Test-of-Cure.

*For key to footnotes, refer to footnotes in Table 5 and below:

Clinical outcome assessment and collection of a sample for urine culture was completed on Day 7.

**Investigator-Determined Clinical Response Categories at the EOT (IV), TOC, and LFU Visits**

The primary efficacy endpoint was the proportion of patients with an overall success (clinical cure and microbiologic eradication) in the m-MITT Population at the TOC Visit. Overall responses were programmatically determined and defined as follows:

- Overall success: a patient deemed a clinical cure AND who achieved microbiologic eradication.
- Overall failure: a patient deemed a clinical failure OR who was deemed to have microbiologic persistence.
- Overall indeterminate: insufficient data were available to determine if the patient was an overall success or failure.

**Determination of Overall Response**

| Microbiologic Response | Clinical Response | Overall Response |
| --- | --- | --- |
| Eradication | Cure | Success |
| Eradication | Failure | Failure |
| Eradication | Indeterminate | Indeterminate |
| Persistence | Cure | Failure |
| Persistence | Failure | Failure |
| Persistence | Indeterminate | Failure |
| Indeterminate | Cure | Indeterminate |
| Indeterminate | Failure | Failure |
| Indeterminate | Indeterminate | Indeterminate |

**Clinical Outcomes Definitions**

Based on the assessment of signs and symptoms, the Investigator chose 1 of the following clinical outcomes at the Day 5, EOT, and TOC Visits:

- Clinical cure: complete resolution or significant improvement of signs and symptoms of cUTI or AP that were present at Baseline and no new symptoms, such that no further antimicrobial therapy was warranted.

For outcome at Day 5 or EOT, if a patient discontinued study drug due to an adverse event and did not receive non-study antibacterial therapy for the cUTI or AP, the patient was considered a clinical cure.

- Clinical failure: symptoms of cUTI or AP present at study entry had not completely resolved or new symptoms developed and required the initiation of non-study antibacterial therapy, or death.

For outcome at Day 5 or EOT, if a patient discontinued study drug due to an adverse event, and received non-study antibacterial therapy for the cUTI or AP, the patient was considered a clinical failure.

- Clinical indeterminate: insufficient data were available to determine if the patient was a cure or failure.

Based on the assessment of signs and symptoms, the Investigator chose 1 of the following clinical outcomes at the LFU Visit:

- Sustained clinical cure: met criteria for clinical cure at the TOC and remained free of signs and symptoms of cUTI or AP at the LFU Visit.
- Relapse: clinical cure at the TOC visit but new signs and symptoms of cUTI were present at the LFU Visit, and the patient required antibiotic therapy for the cUTI.
- Clinical indeterminate: insufficient data were available to determine if the patient was a sustained clinical cure or clinical relapse.

Note: If a patient was a clinical failure at EOT, the patient was automatically considered a failure at the TOC and LFU Visits, and an assessment of clinical response by the Investigator was listed as “failure at EOT.”

**Microbiological Response Categories at the EOT (IV), TOC, and LFU Visits for Each Pathogen Identified at Baseline**

Per-patient microbiologic response was determined programmatically based on the results of blood and urine cultures as 1 of the following outcomes at the Day 5, EOT, and TOC Visits:

- Microbiologic eradication: the demonstration that the baseline bacterial pathogen(s) was reduced to <10^4^ CFU/mL on urine culture and negative on repeat blood culture (if positive at Baseline).
- Microbiologic persistence: the urine culture grew ≥10^4^ CFU/mL of any of the baseline pathogen(s) identified at study entry and/or a blood culture demonstrated the same baseline pathogen(s). Patients who were a persistence at EOT were considered a persistence at TOC.
- Microbiologic indeterminate: no follow-up urine culture was available, the follow-up urine culture could not be interpreted for any reason, or the follow-up urine culture was considered contaminated.

Per-patient microbiologic response was determined programmatically based on the results of blood and urine cultures as 1 of the following outcomes at the LFU Visit:

- Sustained microbiologic eradication: microbiologic eradication at the TOC and LFU Visits.
- Presumed sustained microbiologic eradication: no urine culture was done at LFU, and the patient met clinical criteria for sustained clinical cure.
- Microbiologic recurrence: urine culture grew ≥10^4^ CFU/mL of any of the baseline pathogen(s) identified at study entry and/or a positive blood culture at any time after documented eradication at the TOC Visit up to and including the LFU Visit.
- Microbiologic indeterminate: no follow-up urine culture was available, the follow-up urine culture could not be interpreted for any reason, or the follow-up urine culture was considered contaminated.

##### **Additional Microbiologic Outcomes**

- Colonization: the isolation of a new pathogen(s) at ≥10^5^ CFU/mL (other than the original baseline pathogen[s]) from a urine culture in a patient who was assessed as a clinical cure.
- Superinfection: the isolation of a new pathogen(s) at ≥10^5^ CFU/mL (other than the original baseline pathogen[s]) from a urine culture that was accompanied by clinical signs and symptoms of infection requiring alternative antimicrobial therapy (ie, the patient was assessed by the Investigator as a clinical failure) during the period up to and including EOT.
- New infection: the isolation of a new pathogen(s) at ≥10^5^ CFU/mL (other than the original baseline pathogen[s]) from a urine culture that was accompanied by clinical signs and symptoms of infection requiring alternative antimicrobial therapy (ie, the patient was assessed by the Investigator as a clinical failure) in the time period after EOT.

**Analysis Populations**

- The Intent-to-Treat (ITT) Population included all patients randomized to study drug treatment (ZTI 01 or PIP-TAZ) regardless of whether the patient actually received study drug.
- The MITT Population included patients who met ITT criteria and received any amount of study drug.
- The m­MITT Population included patients who met MITT criteria and had ≥1 baseline Gram negative pathogen from an appropriately collected pre-treatment baseline urine or blood sample.
- The CE Populations (CE-EOT, CE­TOC, and CE-LFU) included patients who met inclusion and exclusion criteria, received ≥9 doses of study drug, had the EOT, TOC, LFU Visits occur within the window, and did not have an indeterminate clinical response at the specified visit.
- The ME Populations (ME-EOT, ME-TOC, and ME-LFU) included patients who met m-MITT criteria and CE criteria and had an appropriately collected urine culture specimen and interpretable urine culture result at the EOT, TOC, and LFU Visits, respectively.
- The Safety Population included patients who met ITT criteria and received any amount of study drug. All safety analyses were based on actual treatment received.
- The PK Population included patients who received ≥1 dose of ZTI-01 and had ≥1 quantifiable ZTI 01 plasma concentration available for analysis.

Supplementary Appendix Table 2. Overall Response at Test-of-Cure with Baseline Pathogens Resistant, Susceptible, or Intermediate to Piperacillin-Tazobactam at Baseline (Population: Microbiologic Modified Intent-to-Treat)

| Pathogens Resistant or Susceptible to Piperacillin-Tazobactam  Overall Response | ZTI-01 | PIP-TAZ | Treatment Comparison | |
| --- | --- | --- | --- | --- |
|  | N = 184  n (%) | N = 178  n (%) | Difference (%) | 95% CI |
| Susceptible, N | 153 | 150 |  |  |
| Success | 99 (64.7) | 85 (56.7) | 8.0 | (-3.6, 19.7) |
| Failure/Indeterminate |  |  |  |  |
| Failure | 44 (28.8) | 58 (38.7) |  |  |
| Indeterminate | 10 (6.5) | 7 (4.7) |  |  |
| Resistant, N | 14 | 9 |  |  |
| Success | 11 (78.6) | 3 (33.3) | 45.2 | (-1.4, 91.9) |
| Failure/Indeterminate |  |  |  |  |
| Failure | 3 (21.4) | 6 (66.7) |  |  |
| Intermediate, N | 12 | 8 |  |  |
| Success | 7 (58.3) | 5 (62.5) | -4.2 | (-58.2, 49.9) |
| Failure/Indeterminate |  |  |  |  |
| Failure | 5 (41.7) | 3 (37.5) |  |  |
| Unknown, N | 5 | 11 |  |  |
| Success | 2 (40.0) | 4 (36.4) | 3.6 | (-62.4, 69.7) |
| Failure/Indeterminate |  |  |  |  |
| Failure | 2 (40.0) | 6 (54.5) |  |  |
| Indeterminate | 1 (20.0) | 1 (9.1) |  |  |

Percentages were calculated using N, the number of patients in the subgroup as the denominator.

Susceptible (MIC ≤16 µg/mL), intermediate susceptible (MIC 32 ug/mL to 64 ug/mL), and resistant (MIC ≥128 ug/mL) were determined using CLSI breakpoints for Enterobacteriaceae for PIP-TAZ.

Treatment difference was the difference in the overall success rate between the 2 treatment groups (ZTI 01 - PIP-TAZ). The 95% CIs (2-sided) were computed using a continuity-corrected Z-statistic.

Overall success was defined as Clinical cure and microbiologic eradication.

CI: confidence interval; CLSI: Clinical and Laboratory Standards Institute; MIC: minimum inhibitory concentration; PIP: piperacillin; TAZ: tazobactam.
